# Remote magnetic navigation shows superior long-term outcomes in pediatric atrioventricular (nodal) tachycardia ablation compared to manual radiofrequency and cryoablation

**DOI:** 10.1016/j.ijcha.2021.100881

**Published:** 2021-10-01

**Authors:** Anna M.E. Noten, Janneke A.E. Kammeraad, Nawin L. Ramdat Misier, Sip Wijchers, Ingrid M. van Beynum, Michiel Dalinghaus, Thomas B. Krasemann, Sing-Chien Yap, Natasja M.S. de Groot, Tamas Szili-Torok

**Affiliations:** a Department of Cardiology, Erasmus MC, University Medical Center, Rotterdam, the Netherlands^1^; b Department of Pediatric Cardiology, Sophia Children’s Hospital, Erasmus MC, University Medical Center, Rotterdam, the Netherlands^1^

**Keywords:** Catheter ablation, Remote magnetic navigation, Cryoablation, Radiofrequency ablation, Supraventricular tachycardia, Atrioventricular reentry tachycardia, Atrioventricular nodal reentry tachycardia, Pediatrics, **AP**, accessory pathway, **AVNRT**, atrioventricular nodal reentry tachycardia, **AVRT**, atrioventricular reentry tachycardia, **CA**, catheter ablation, **CHD**, congenital heart defect, **CRYO**, cryoablation, **DAP**, dose area product, **EAM**, electro-anatomic mapping, **ECG**, electrocardiogram, **MAN**, manual, **RF**, radiofrequency, **RMN**, remote magnetic navigation, **SVT**, supraventricular tachycardia

## Abstract

**Background:**

Catheter ablation (CA) is the first-choice treatment for tachyarrhythmia in children. Currently available CA techniques differ in mechanism of catheter navigation and energy sources. There are no large studies comparing long-term outcomes between available CA techniques in a pediatric population with atrioventricular reentry tachycardia (AVRT) or atrioventricular nodal reentry tachycardia (AVNRT) mechanisms.

**Objective:**

This study aimed to compare procedural and long-term outcomes of remote magnetic navigation-guided radiofrequency (RF) ablation (RMN), manual-guided RF ablation (MAN) and manual-guided cryoablation (CRYO).

**Methods:**

This single-center, retrospective study included all first consecutive CA procedures for AVRT or AVNRT performed in children without structural heart disease from 2008 to 2019. Three study groups were defined by the ablation technique used: RMN, MAN or CRYO. Primary outcome was long-term recurrence of tachyarrhythmia.

**Results:**

In total, we included 223 patients, aged 14 (IQR 12–16) years; weighting 56 (IQR 47–65) kilograms. In total, 108 procedures were performed using RMN, 76 using MAN and 39 using CRYO. RMN had significantly lower recurrence rates compared to MAN and CRYO at mean follow-up of 5.5 ± 2.9 years (AVRT: 4.3% versus 15.6% versus 54.5%, P < 0.001; AVNRT: 7.7% versus 8.3% versus 35.7%, P = 0.008; for RMN versus MAN versus CRYO respectively). In AVNRT ablation, RMN had significantly lower fluoroscopy doses compared to CRYO [30 (IQR 20–41) versus 45 (IQR 29–65) mGy, P = 0.040).

**Conclusion:**

In pediatric patients without structural heart disease who underwent their first AV(N)RT ablation, RMN has superior long-term outcomes compared to MAN and CRYO, in addition to favorable fluoroscopy doses.

## 1 Introduction

In children without structural heart disease, the majority of supraventricular tachyarrhythmias (SVT) consist of atrioventricular nodal reentrant tachycardia (AVNRT) and atrioventricular reentrant tachycardia (AVRT) [1], [2], [3]. Catheter ablation (CA) in pediatric cardiology has been used as treatment of these SVT since 1990 [1], [4]. Over the years, the indication of CA in pediatric patients has evolved from treatment of drug refractory tachyarrhythmia only, to a first-line treatment and patient preference [1], [5].

Initially, large-scale registries were conducted which reported favorable success and complication rates of manual guided radiofrequency (RF) ablation in children [1], [4], [6]. Comparable to the adult population, other CA techniques also became available in pediatric cardiology over time. Manual guided cryoablation and remote magnetic navigation (RMN) guided RF ablation were first used in children in 2003 [7], [8] and 2009 [9], [10], [11] respectively and became standard-of-care treatment options. Theoretically, these techniques might have advantages compared to the manual RF technique. Cryothermal energy allows cryomapping (with reversible effect) before establishing a permanent lesion. Furthermore the cryocatheter’s tip adheres to the myocardium during energy application. These could be beneficial, especially when targeting the proximity of vulnerable cardiac structures such as the His bundle [8], [12]. With respect to RMN-guided RF ablation, the ablation catheter has a flexible, magnetically enabled distal tip and is navigated toward and held to the ablation surface by magnetic force, facilitating an omnidirectional positioning of the catheter with high stability [13]. Prior reports demonstrated improved lesion formation and superior safety compared to manual RF ablation [14], [15], which also could be very beneficial in treatment of younger and smaller patients [9], [11].

In the pediatric population, several small studies were conducted and reported first experiences with the mentioned techniques [2], [3], [8], [9], [11], [16], [17]. There are no large scale studies comparing ablation techniques in children, nor investigating long-term outcomes. Hence, this study aimed to compare procedural- and long-term outcomes of three ablation techniques - RMN-guided RF ablation, manual-guided RF ablation and manual-guided Cryoablation - in a large group of children diagnosed with AVRT and AVNRT with a structural normal heart. We hypothesized that ablative therapy of AV(N)RT guided by RMN or cryothermal energy has improved short- and long-term outcomes compared to manual RF ablation.

## 2 Methods

### 2.1 Study design

This single-center, retrospective study investigated all children with a structural normal heart, undergoing their first CA procedure for AVNRT and AVRT. Outcomes were compared between three groups, based on the ablation technique used: RMN-guided RF ablation (RMN), manual guided RF ablation (MAN), and manual guided cryoablation (CRYO), for each of the two SVT mechanisms separately. Primary endpoint was documented recurrence of tachyarrhythmia and/or (if present pre-procedurally) recurrence of pre-excitation on 12-lead ECG during long-term follow-up. We also analyzed the following secondary endpoints: procedural parameters, acute procedural success, the need for repeat procedures and complication rates. The study protocol conforms to the ethical guidelines of the 1975 Declaration of Helsinki. Procedural informed consent was obtained from all the patients prior to the EPS. The local medical ethics committee determined this study was not subject to the Dutch Medical Research Involving Human Subjects Act (WMO). The need for individual consent was waived by the local medical ethical committee.

### 2.2 Study population

All consecutive patients, aged < 18 years, undergoing their first CA procedure for AVNRT or AVRT mechanisms between the 1st of January 2008 until 30th of June 2019, were included in this study. Patients were included from a single, high-volume CA center with large experience with all available CA techniques, both in adults as in pediatrics. Redo procedures were excluded. Patients had either documented SVT or symptoms of palpitations or syncope with documented pre-excitation on a 12-lead ECG. We excluded patients in whom cross-over between CA ablation techniques during the procedure occurred and patients with any types of congenital heart defect (CHD).

### 2.3 Definitions

Total procedure time was defined as the time from first skin puncture until the removal of sheaths. The endpoints of acute procedural success were defined as the elimination of accessory pathway (AP) conduction for AVRT and non-inducibility of the tachycardia (i.e. not even a single echo beat was accepted) for AVNRT. Recurrence was defined as an episode of sustained SVT recorded on 12-lead ECG, 24-hour to 7-day continuous rhythm observation and/or implantable loop recorder or pacemaker or ICD. Minor complications were access site complications, temporary bundle branch block or AV block, pericardial effusion not requiring intervention. Major complications were permanent second- or third-degree atrioventricular block, cardiac tamponade, hemorrhagic shock, stroke and procedure-related death. Access site complications were vessel wall and bleeding complications which required surgical intervention or intervention by radiologist, prolonged hospitalization and/or an Hb drop of > 1.8 mmol/L.

### 2.4 Data collection

Baseline demographic and clinical characteristics were collected from the institutional electronic patient dossier [HiX version 6.1 (ChipSoft BV, Amsterdam, NL)]. Procedural data was derived both from the electronic medical files, as well as from the electronic procedural log files recorded with EP-workmate (St. Jude Medical Inc., St. Paul, MN, USA), CryoConsole (Medtronic, Minneapolis, MN, USA) and Odyssey Cinema (Stereotaxis Inc., St. Louis MO, USA) systems. All patient information was de-identified.

### 2.5 Procedural protocol

All antiarrhythmic drugs were discontinued for > 5 half-lives before the procedure. All procedures were performed under general anesthesia. Vascular access was attained with placement of sheaths and diagnostic catheters in left and/or right femoral vein and/or artery, with sheath sizes and catheter types varying based on operator preference. The choice between a transseptal or retrograde aortic approach in case of left‐sided AP was also based on operator preference. A standard EPS was performed with diagnostic catheters positioned in the coronary sinus (CS), right ventricular apex, and in the His position. The diagnosis of dual atrioventricular nodal pathways, atrioventricular nodal reentrant tachycardia, or AP-mediated tachycardia was made using standard diagnostic criteria. Regarding AVNRT ablation, Koch’s triangle was mapped by bending and pulling the ablation catheter from the His position and rotating to the coronary sinus in order to obtain a slow pathway potential with a small preferably fractionated atrial and large ventricular electrogram. Ablation was applied at this site in order to eliminate slow pathway conduction. Appearance of junctional ectopic rhythm was used to guide RF delivery. Ablation was performed using the following RF settings: AVNRT: 10 W gradually increasing to 45 W, non-irrigated, maximum 55 °C; AVRT: 55–60 W, non-irrigated, maximum 50–60 °C, for 60 s. In AVNRT, the procedural endpoint was non-inducibility of the tachycardia (i.e. not even a single echo beat was accepted). In case of empirical slow pathway ablation, the procedural endpoint was elimination of dual AV nodal conduction. Inducibility was tested after each application. In AVRT, the endpoint of the procedure was persistent absence of AP conduction. Testing with isoprenaline and/or adenosine was performed based on operator’s preference. A standard of 30 min waiting time was applied to all procedures. Procedures were performed by the same team of operators over time.

In the RMN group, procedures were performed using the Stereotaxis Niobe system (Stereotaxis, Inc., St Louis, MO, USA). The following ablation catheters were used in RMN: Celsius RMT, ThermoCool RMT (Biosense Webster, Diamond Bar, CA, USA), Trignum Flux gold tip (Biotronik GmbH, Berlin, Germany) or Magnoflush gold tip (MedFact Engineering GmbH, Lorrach, Germany). The procedures in the MAN group were performed using the following ablation catheters: Celcius or ThermoCool (Biosense Webster, Diamond Bar, CA, USA). Cryoablation was performed using Freezor 4 mm (before 2011) and 6 mm (from 2011 until 2019) catheters (Medtronic Inc, Minneapolis, MN, USA).

### 2.6 Statistical analysis

Normality was assessed by the Kolmogorov-Smirnov test, or when appropriate, Shapiro-Wilk test. Mean and standard deviation (SD) were calculated for normally distributed continuous variables. Median, interquartile range (IQR) and range were computed for continuous variables with non-normal distribution. Descriptive statistics for categorical data were expressed in absolute numbers and percentages. Normally distributed continuous variables were analyzed using the 1-way ANOVA. For continuous variables with non-normal distributions, the Kruskall-Wallis test was used. Post-hoc pairwise comparisons were performed using the Dunn-Bonferroni approach, when a significant main effect was present. For comparing frequencies, the Chi-square test was used, or, when appropriate, Fisher's exact test. Univariable and Multivariable Cox proportional hazards models were used to examine the relationship between treatment group and long-term outcomes, adjusting for potential confounders. A 2-sided P-value of < 0.05 was considered significant. Data were analyzed using SPSS 26.0 (SPSS Inc., Chicago, IL, USA).

## 3 Results

In total, 293 procedures were performed in 247 children within the mentioned time frame. We identified 247 (84.3%) first procedures and 46 (15.7%) redo procedures, of which the latter were excluded. Eleven patients (4.5%) with CHD of varying severity and 13 patients (5.3%) in whom cross-over between different techniques occurred were also excluded. Accordingly, analysis in 223 patients was performed. Seventy-nine patients were diagnosed with AVNRT and 144 with AVRT. In total, 108 (48.4%) patients were treated with RMN, 76 (34.1%) were treated with MAN and 39 (17.5%) with CRYO ablation technique. The utilization of the various techniques over time is presented as supplemental Fig. 1.

### 3.1 Baseline demographic and clinical data

Baseline demographic and clinical data is presented in Table 1. AVNRT patients had a median age of 15 (IQR 12–16; Range 5–18) years, whereas AVRT patients had a median age of 14 (IQR 12–16; Range 6–17) years. The median weight in the AVNRT subgroup was 59 (IQR 48–66; Range 20–88) kilograms and in AVRT 56 (IQR 45–65; Range 23 –91) kilograms. (Supplemental Fig. 2). The majority of patients experienced palpitations (AVNRT 100.0%, AVRT 90.9%), however a small portion of AVRT patients also had an event of syncope (7.7%). Fourteen children with AVRT (9.7%) were asymptomatic and a pre-excitation on a 12-lead ECG was accidentally found at a medical check-up (e.g. related to sport, school or profession). Baseline demographic and clinical data were comparable between groups, except for sotalol being less frequently used in AVRT ablation in the RMN group (14.5%, compared to 18.8% in MAN and 54.5% in CRYO, P = 0.008).

**Table 1:** Baseline demographic and clinical data.

**AVNRT ablation**	RMN N = 39	MAN N = 12	CRYO N = 28	Total N = 79	P-value
Female	22 (56.4%)	7 (58.3%)	16 (57.1%)	45 (57.0%)	0.99
Age (year)	15 (12–16)	15 (12–17)	14 (11–16)	15 (12–16)	0.53
Weight (kg)	62 (48–67)	60 (49–67)	53 (43–64)	59 (48–66)	0.25
Length (cm)	169 (161–177)	167 (158–172)	164 (154–175)	166 (158–175)	0.33
BMI (kg/m^2^)	21 (19–23)	22 (18–23)	19 (17–21)	20 (18–22)	0.19
Palpitations	39 (100.0%)	12 (100.0%)	28 (100.0%)	79 (100.0%)	ns
Syncope	1 (2.6%)	0 (0.0%)	1 (3.6%)	2 (2.5%)	0.81
Family History Arrhythmias	3 (7.7%)	0 (0.0%)	2 (7.1%)	5 (6.3%)	0.62
Family History Sudden death	1 (2.6%)	1 (8.3%)	0 (0.0%)	2 (2.5%)	0.31
*Medication*					
Beta-blocker	1 (2.6%)	0 (0.0%)	2 (7.1%)	3 (3.8%)	0.47
Sotalol	6 (15.4%)	3 (25.0%)	6 (21.4%)	15 (19.0%)	0.70
Digoxine	0 (0.0%)	0 (0.0%)	0 (0.0%)	0 (0.0%)	ns
Verapamil	2 (5.1%)	0 (0.0%)	3 (10.7%)	5 (6.3%)	0.40
Flecainide	0 (0.0%)	1 (8.3%)	0 (0.0%)	1 (1.3%)	0.06

**AVRT ablation**	RMN N = 69	MAN N = 64	Cryo N = 11	Total N = 144	P-value
Female	31 (44.9%)	31 (48.4%)	4 (36.4%)	66 (45.8%)	0.74
Age (year)	14 (12–16)	15 (12–17)	14 (11–16)	14 (12–16)	0.48
Weight (kg)	53 (42–65)	57 (49–65)	53 (43–64)	56 (45–65)	0.65
Length (cm)	165 (155–177)	170 (158–180)	164 (154–175)	167 (156–177)	0.44
BMI (kg/m^2^)	19 (18–22)	20 (18–21)	19 (17–21)	19 (18–21)	0.98
Palpitations	62 (89.9%)	57 (90.5%)	11 (100.0%)	130 (90.9%)	0.55
Syncope	6 (8.7%)	4 (6.3%)	1 (9.1%)	11 (7.7%)	0.87
Asymptomatic*	6 (8.7%)	8 (12.5%)	0 (0.0%)	14 (9.7%)	0.40
Family History Arrhythmias	11 (16.2%)	5 (7.9%)	2 (18.2%)	18 (12.7%)	0.31
Family History Sudden death	5 (7.5%)	2 (3.2%)	0 (0.0%)	7 (5.0%)	0.39
*Medication*					
Beta-blocker	2 (2.9%)	1 (1.6%)	1 (9.1%)	4 (2.8%)	0.37
Sotalol	10 (14.5%)	12 (18.8%)	6 (54.5%)	28 (19.4%)	**0.008**
Digoxine	1 (1.4%)	0 (0.0%)	0 (0.0%)	1 (0.7%)	0.58
Verapamil	5 (7.2%)	0 (0.0%)	1 (9.1%)	6 (4.2%)	0.08
Flecainide	1 (1.4%)	0 (0.0%)	0 (0.0%)	1 (0.7%)	0.58

BMI: body mass index, CRYO: manual guided cryoablation, MAN: manual-guided radiofrequency ablation, RMN: remote magnetic navigation guided radiofrequency ablation.

* Asymptomatic patients in whom accidentally a WPW pattern on 12-lead ECG was discovered at a (e.g. sport or profession related) regular medical check-up.

### 3.2 Procedural outcome

Standard EPS revealed AVNRT mechanism in 79 (35.4%) patients and AP-mediated tachycardia (AVRT) in 144 (64.6%) patients (Table 2). With respect to left-sided AP’s, a transseptal approach was used in 5.2% of patients whereas a retrograde aortic approach was used in 94.8%. The majority of AVNRTs were typical with slow-fast AVNRTs (slow-fast: 88.6%, slow-slow: 2.5%, fast-slow: 3.8%) (Table 2). Most AVRTs had a Wolff-Parkinson-White (WPW) pattern on 12-lead ECG with antegrade conduction over the AP (59.7%), whereas in 40.3% a concealed bypass was found (Table 2).

**Table 2:** Procedural parameters.

**AVNRT ablation**	RMN N = 39	MAN N = 12	CRYO N = 28	Total N = 79	P-value
*Type*					
Typical (slow-fast)	33 (84.5%)	11 (91.7%)	26 (92.9%)	70 (88.6%)	0.54
Atypical (slow–slow)	1 (2.6%)	0 (0.0%)	1 (3.6%)	2 (2.5%)	0.81
Atypical (fast–slow)	2 (5.1%)	0 (0.0%)	1 (3.6%)	3 (3.8%)	0.72
Empirical slow pathway ablation	3 (7.7%)	1 (8.3%)	0 (0.0%)	4 (5.1%)	0.31
*Parameters*					
Procedure time (min)	101 (87–121)*	88 (62–99)*	120 (88–143)*	101 (85–128)	**0.018**
Fluoroscopy dose (mGy)	30 (20–41)**	27 (17–77)**	45 (29–65)**	33 (21–51)	**0.041**
Ablation time (sec)	193 (104–322)***	352 (88–711)***	572 (480–932)***	279 (118–609)	**0.001**
Application number	5 (2–10)	3 (2–10)	3 (2–5)	4 (2–7)	0.15
*Acute success*					
Acute success	39 (100.0%)	12 (100.0%)	24 (85.7%)	75 (94.9%)	**0.022**

**AVRT ablation**	RMN N = 69	MAN N = 64	Cryo N = 11	Total N = 144	P-value
*Type*					
Concealed AP	26 (37.7%)	24 (37.5%)	8 (72.7%)	58 (40.3%)	0.07
WPW	43 (62.3%)	40 (62.5%)	3 (27.3%)	86 (59.7%)	0.07
*AP Location*					
LA free wall	46 (66.7%)	36 (56.3%)	0 (0.0%)	82 (56.9%)	**<0.001**
RA free wall	8 (11.6%)	3 (4.7%)	0 (0.0%)	11 (7.6%)	0.20
Septal	13 (18.8%)	20 (31.2%)	3 (27.3%)	36 (25.0%)	0.25
Parahisian	2 (2.9%)	0 (0.0%)	7 (63.6%)	9 (6.3%)	**<0.001**
Multiple AP	0 (0.0%)	5 (7.8%)	1 (9.1%)	6 (4.2%)	0.06
*Parameters*					
Procedure time (min)	105 (88–135)	100 (81–160)	150 (107–183)	105 (83–143)	0.15
Fluoroscopy dose (mGy)	42 (26–61)	55 (32–85)	57 (39–98)	48 (30–81)	0.07
Ablation time (sec)	157 (78–365)	195 (74–409)	480 (300–691)	180 (78–415)	0.05
Application number	3 (1–9)	5 (2–12)	2 (2–6)	4 (2–10)	0.36
*Acute success*					
Acute success	68 (98.6%)	61 (95.3%)	9 (81.8%)	138 (95.8%)	**0.035**
Disappearance of pre-excitation on ECG (if present pre-procedural)	41 (97.6%)	39 (95.1%)	3 (100.0%)	83 (96.5%)	0.78

Post-hoc pairwise comparison using the Dunn-Bonferroni approach:

* Procedure time: RMN versus MAN P = 0.028; MAN versus CRYO P = 0.005; RMN versus CRYO P = 0.029.

** Fluoroscopy dose: RMN versus MAN P = 0.71; MAN versus CRYO P = 0.17; RMN versus CRYO P = 0.012.

*** Ablation time: RMN versus MAN P = 0.37; MAN versus CRYO P = 0.12; RMN versus CRYO P < 0.001.

AP: accessory pathway, AVNRT: atrioventricular nodal reentry tachycardia, AVRT: atrioventricular reentry tachycardia, CRYO: manual guided cryoablation, LA: left atrium, MAN: manual-guided radiofrequency ablation, RA: right atrium, RMN: remote magnetic navigation guided radiofrequency ablation, WPW: Wolff-Parkinson-White

**Fig. 1:**
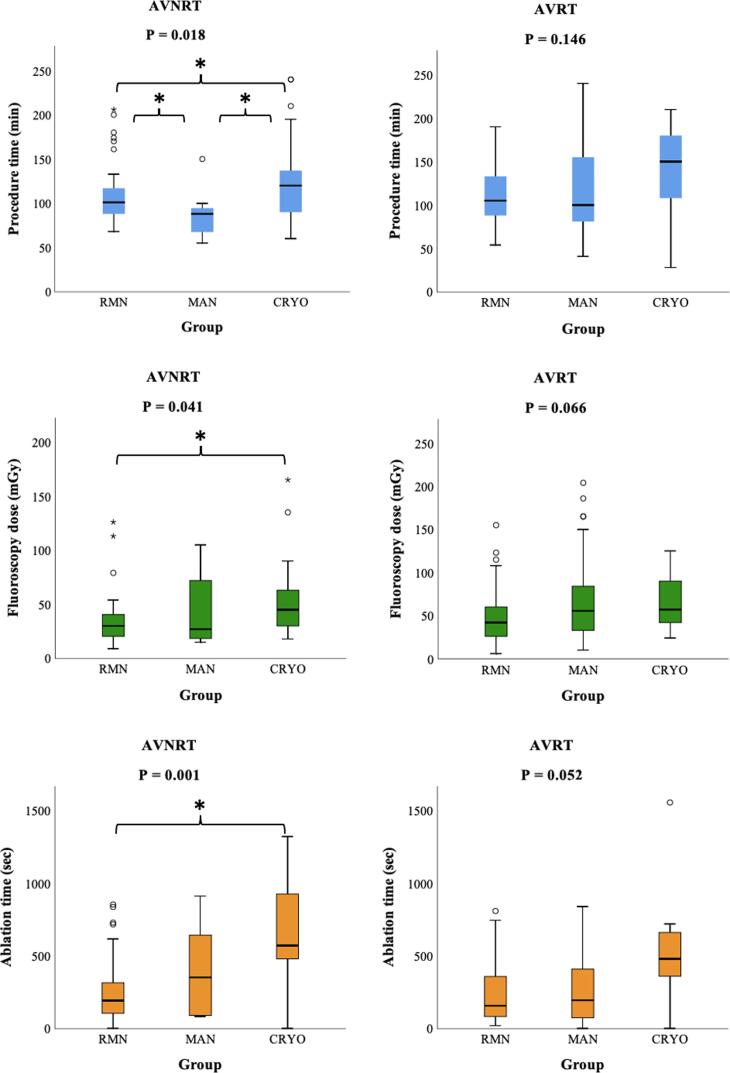
Procedural parameters. Mean and interquartile range of three procedural parameters: procedure time, fluoroscopy dose and ablation time. Significant results of the Post-hoc pairwise comparison using the Dunn-Bonferroni approach are marked with an asterisk (*): AVNRT procedure time: RMN versus MAN P = 0.028; MAN versus CRYO P = 0.005; RMN versus CRYO P = 0.029. AVNRT Fluoroscopy dose: RMN versus CRYO P = 0.012. AVNRT Ablation time: RMN versus CRYO P < 0.001. AVNRT: atrioventricular nodal reentry tachycardia, AVRT: atrioventricular reentry tachycardia, CRYO: manual guided cryoablation, MAN: manual-guided radiofrequency ablation, RMN: remote magnetic navigation guided radiofrequency ablation.

In AVNRT, procedure times were shortest in MAN procedures [88 (IQR 62–99) minutes], then in RMN [101 (IQR 87–121) minutes] and longest in CRYO [120 (IQR 88–143) minutes] (P = 0.018) (Table 2 and Fig. 1). Post-hoc pairwise comparison showed a significant difference between all three groups [MAN versus RMN (P = 0.028); MAN versus CRYO (P = 0.005); RMN versus CRYO (P = 0.029)]. Fluoroscopy dose was significantly different between groups [RMN 30 (IQR 20–41), versus MAN 27 (IQR 17–77), versus CRYO 45 (IQR 29–65) mGy, P = 0.041]. Post-hoc analysis showed that this consisted of a significant difference between RMN compared to CRYO (P = 0.012), whereas RMN versus MAN (P = 0.71) and MAN versus CRYO (P = 0.17) were comparable. Ablation time was significantly shorter in RMN compared to CRYO [RMN 193 (IQR 104–322), MAN 352 (IQR 88–711), CRYO 572 (IQR 480–932) seconds, P = 0.001; post-hoc analysis: RMN versus CRYO P < 0.001, RMN versus MAN P = 0.37 and MAN versus CRYO P = 0.12)]. We found a 100.0% acute success rate in RMN and MAN, compared to 85.7% in CRYO (P = 0.022).

Regarding AVRT, procedure times were comparable between groups (Table 2 and Fig. 1). We observed a lower fluoroscopy dose and ablation time in RMN, however this did not reach the level of statistical significance [fluoroscopy dose: 42 (IQR 26–61) versus 55 (IQR 32–85), versus 57 (IQR 39–98) mGy, for RMN versus MAN versus CRYO respectively, P = 0.07] [ablation time: 157 (IQR 78–365) versus 195 (IQR 74–904) versus 480 (IQR 300–691) seconds, for RMN versus MAN versus CRYO respectively, P = 0.05)]. Similar to AVNRT ablation, we observed significantly higher acute success rates in RMN and MAN, compared to CRYO (98.6% versus 95.3% versus 81.8% respectively, P = 0.035).

### 3.3 Long-term outcome

**Fig. 2:**
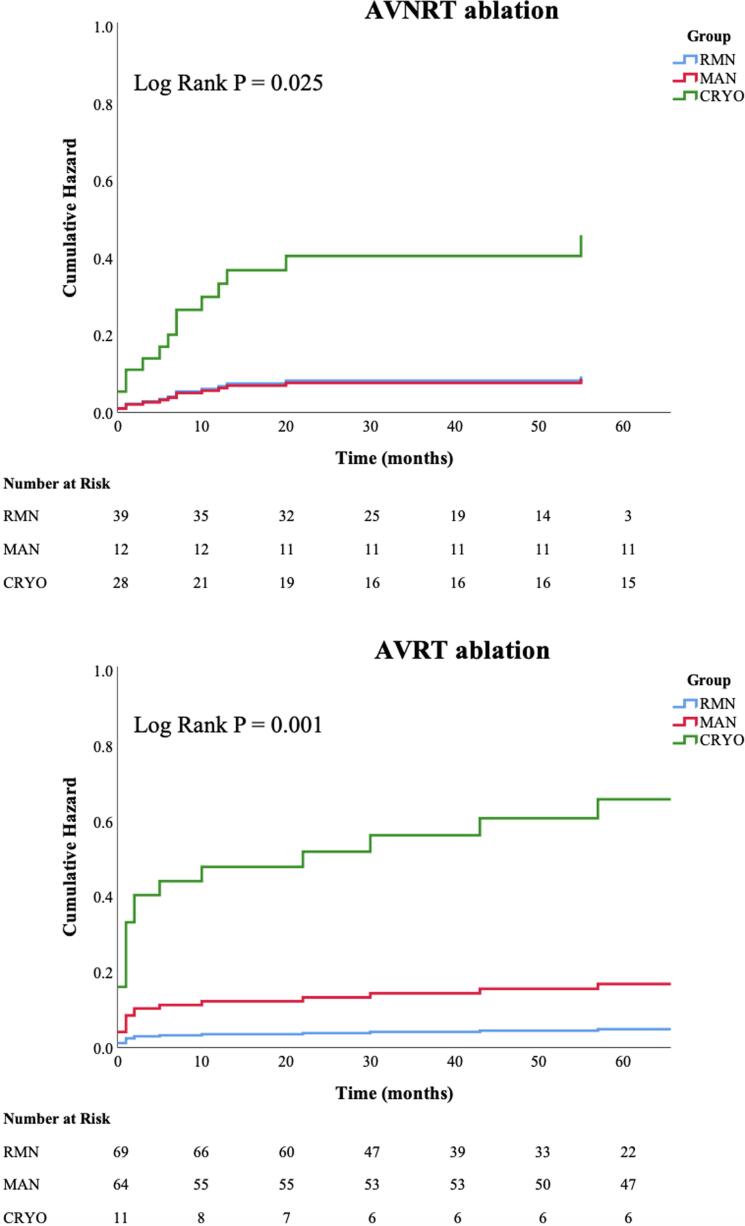
Recurrence rates - cumulative hazard on arrhythmia recurrence. Display of the cumulative hazard on atrioventricular nodal reentry tachycardia (AVNRT) - and atrioventricular reentry tachycardia (AVRT) recurrence, compared between three techniques: remote magnetic navigation guided radiofrequency ablation (RMN), manual guided radiofrequency ablation (MAN) and manual guided cryoablation (CRYO).

Regarding AVNRT ablation, we observed significantly lower recurrence rates in RMN and MAN guided ablation, compared to CRYO (7.7% versus 8.3% versus 35.7% respectively, P = 0.008) (Table 3). The mean follow-up time was 5.5 ± 2.9 years. AVNRT procedures performed with RF (i.e. MAN and RMN) were associated with a lower risk of recurrence during the follow-up, when compared to CRYO (Log-Rank P = 0.025; For RMN: HR 0.20, 95%-CI 0.06–0.74; For MAN: HR 0.19, 95%-CI 0.02–1.48; with CRYO as the reference group) (Fig. 2). As a sensitivity analysis, age, gender and weight were consecutively also added to the models, and did not show any significant associations with the primary outcome (data not shown).

**Table 3:** Long-term outcomes.

**AVNRT**	RMN N = 39	MAN N = 12	CRYO N = 28	Total N = 79	P-value
Recurrence	3 (7.7%)	1 (8.3%)	10 (35.7%)	14 (17.7%)	**0.008**
Time to Recurrence (mo)	2 (1–2)	12 (12–12)	6 (1–13)	6 (1–12)	0.49
Recurrence of other arrhythmia	2 (5.1%)	1 (8.3%)	1 (3.6%)	4 (5.1%)	0.82
Redo	2 (5.1%)	1 (8.3%)	9 (32.1%)	12 (15.2%)	**0.008**

**AVRT**	RMN N = 69	MAN N = 64	CRYO N = 11	Total N = 144	P-value
Recurrence	3 (4.3%)	10 (15.6%)	6 (54.5%)	19 (13.2%)	**<0.001**
Time to recurrence (mo)	16 (1–88)	1 (0–29)	6 (1–41)	2 (1–30)	0.33
Recurrence of other arrhythmia	3 (4.3%)	1 (1.6%)	0 (0.0%)	4 (2.8%)	0.52
Redo	5 (7.2%)	9 (14.1%)	5 (45.5%)	19 (13.2%)	**0.002**

AVNRT: atrioventricular nodal reentry tachycardia, AVRT: atrioventricular reentry tachycardia, CRYO: manual guided cryoablation, MAN: manual-guided radiofrequency ablation, MO: months, RMN: remote magnetic navigation guided radiofrequency ablation

With respect to AVRT ablation, RMN had superior long-term outcomes compared to the other techniques (4.3% versus 15.6% versus 54.5%, respectively P < 0.001), at a mean follow-up of 5.5 ± 2.9 years. The need to perform a repeat procedure showed the same pattern. Median time to recurrence was 6 (IQR 1–12) months in AVNRT and 2 (IQR 1–30) months in AVRT. Regarding AVRT, RMN and MAN groups had a significantly lower risk of recurrence, compared to CRYO (Log-rank P = 0.001; For RMN: HR 0.07, 95%-CI 0.02–0.30; For MAN: HR 0.26, 95%–CI 0.09–0.71; with CRYO as the reference group) (Fig. 2). In addition, RMN had significant lower risk of recurrence compared to MAN (Log-rank P = 0.044; HR 0.29 95%-CI 0.08–1.08, with MAN as the reference group). Age, gender and weight did not show any significant associations with the long-term outcome (data not shown).

### 3.4 Septal and parahisian accessory pathways

APs were predominantly located at the left free wall (56.9%), whereas right free wall, septal and parahisian locations were less frequently observed (Table 2). CRYO was less frequently used for AP’s located at the left free wall (RMN 66.7%, MAN 56.3%, CRYO 0.0%, P < 0.001). In general, recurrence of arrhythmia occurred much more frequently in septal and parahisian AP’s, compared to the other AP locations (overall recurrence rate per location: left free wall 3.7%, right free wall 18.2%, septal 25.0%, parahisian 55.6%, multiple pathways 0.0%, P < 0.001). Subanalysis of septal and parahisian located AP’s only (N = 45) showed that the three techniques were equally frequently used to treat septal and parahisian AP’s [RMN N = 15 (33.3%), MAN N = 20 (44.4%), CRYO N = 10 (22.2%), P = 0.08] (Table 4). The three techniques had comparable acute success rates (93.3% versus 90.0% versus 80.0% respectively, P = 0.57). We observed superior long-term outcomes in favor of RMN in this subgroup, with significantly lower recurrence rates (13.3% versus 30.0% versus 60.0% for RMN versus MAN versus CRYO respectively, P = 0.047). Septal and parahisian located pathways had a significantly higher risk of recurrence when compared to AP’s at other locations (Log-rank P = 0.001; univariable HR 3.09, 95%-CI 1.55–6.17; with non-septal/parahisian AP as the reference group). In a multi-variable Cox regression model including both AP location and ablation technique, RMN had significantly the lowest risk of recurrence when compared to the two other techniques (Log-rank P < 0.001; For RMN: multivariable HR 0.14 95%-CI 0.06–0.37, with CRYO as the reference group; For MAN: multivariable HR 0.32, 95%-CI 0.15–0.69, with CRYO as the reference group; For RMN: multivariable HR 0.45, 95%-CI 0.17–1.23, with MAN as the reference group).

**Table 4:** Subanalysis of ablation of septal and parahisian located AP.

	RMN N = 15	MAN N = 20	CRYO N = 10	Total N = 45	P-value
Acute success	14 (93.3%)	18 (90.0%)	8 (80.0%)	40 (88.9%)	0.57
Recurrence	2 (13.3%)	6 (30.0%)	6 (60.0%)	14 (31.1%)	**0.047**
Redo	1 (6.7%)	5 (25.0%)	5 (50.0%)	11 (24.4%)	**0.047**

AP: accessory pathway, CRYO: manual guided cryoablation, MAN: manual-guided radiofrequency ablation, RMN: remote magnetic navigation guided radiofrequency ablation

### 3.5 Adverse events

We observed one major complication and two minor complications in total, which were comparable between groups [RMN 0 (0.0%), versus MAN 2 (2.6%), versus CRYO 1 (2.6%), P = 0.24]. One patient had a mild aortic valve regurgitation possibly caused by the retrograde transaortic catheter approach and a transient ischemic attack (TIA) 3 months after the procedure. The minor complications were left bundle branch block (N = 1) and temporary AV block (N = 1) which recovered completely. Another patient died during follow-up due to intestinal ischemia due to a genetic defect (not-procedure related).

## 4 Discussion

This is the first study to compare long-term outcomes of pediatric AVNRT and AVRT ablation procedures between three recognized ablation techniques: RMN-guided RF ablation, manual-guided RF ablation and manual-guided Cryoablation. The main finding of our study is that RMN guided ablation has superior long-term outcomes compared to the other techniques.

### 4.1 Efficacy of catheter ablation procedures

This study observed superior efficacy of the RMN-guided ablation technique in treatment of AP-mediated tachycardias in children. Recurrence of arrhythmia and/or preexcitation on 12-lead ECG was observed only in 4% of cases in this group. Only one other study described long-term outcomes of RMN-guided AP ablation in children [9], where fewer recurrences were observed, though this study had a significantly shorter follow-up duration and only included left free wall AP’s. Ablation of left free wall AP’s has a higher success rate compared to other locations, which is likely to be the explanation for the discrepancy with the current study’s findings [2], [18]. Regarding pediatric AVNRT ablation, there are no studies comparing RMN with other ablation techniques. Our study showed significantly higher long-term efficacy in RMN and MAN groups, compared to cryoablation. In the adult population, similar recurrence rates have been reported in RMN and MAN ablation, which were also comparable between these two techniques, corresponding with the current study’s findings [19], [20]. Recently published large European and Japanese pediatric CA registries, including primarily manual-guided RF and few cryoablation procedures, reported much higher 12-month recurrence rates compared to our findings, for AVNRT as well as for AVRT ablation [2], [3]. Moreover, a higher recurrence rate in cryoablation compared to non-irrigated RF ablation was also observed in one of these registries, whereas the other did no comparison between energy sources [2], [3]. Theoretically, the observed differences between cryoablation and RF ablation could be caused by either energy source, catheter stability and/or (mis)identification of the right target. In patients with AVNRT undergoing slow pathway ablation, junctional ectopy during the application of RF energy is a sensitive marker of successful ablation [21], [22]. During cryoablation, usually accelerated junctional tachycardia is not seen and therefore cannot guide lesion delivery [23]. Lack of this feedback is a possible explanation of the inferior performance of cryoablation in slow-pathway modification. However, the current study observed an inferior performance of cryoablation in AVRT ablation as well, where no ectopic feedback is given during ablation. As cryothermal energy allows cryomapping (with reversible effect) before establishing a permanent lesion, this technique sometimes is used to avoid permanent damage of vulnerable cardiac structures if the target lesion lies close to these, in order to avoid complications. Despite this advantage, this technique appeared to be less beneficial, even when used in the most easy approachable anatomy (i.e. AVNRT ablation) [24]. Therefore, we believe that our results are primarily caused by the combination of energy source and catheter stability, with RF having a deeper tissue penetration than cryothermal energy. We postulate that the improved outcome of RMN over MAN in AVRT ablation, is an affirmation of improved catheter stability facilitated by RMN, in addition to the beneficial effects of RF over cryoablation, resulting in highly effective ablations in the RMN group.

### 4.2 Accessory pathway location

AP’s occur at a variety of locations, of which left lateral is the most frequently observed site [18]. Ablation of left lateral located AP’s yields high acute and long-term success rates, whereas right free wall and septal located AP’s are more difficult to ablate [2], [3], [18]. This is consistent with our findings. The reversible effect of cryomapping, before establishing a permanent lesion during cryoablation, makes this ablation technique very appealing when ablating in the proximity of the His bundle. Nevertheless, we observed high recurrence rates in cryoablation of septal and parahisian pathways. Both RF and cryoablation are sensitive to electrode orientation, contact pressure, and convective thermal effects from blood flow over the electrode-tissue interface, influencing lesion formation [25]. Zones of spherical freezing advance are known as isotherms, with higher temperatures occurring at the periphery of an application, also in cryo-mapping ranges (around −30 °C) [26]. Possibly, targets are temporarily paralyzed but not definitively eliminated by this cryo-map effect, resulting in long-term recurrences. On the contrary, in a model with simulated wall motion, RMN-guided RF lesion dimensions were larger compared to manual-guided RF lesions, consistent with greater catheter stability [14]. The improved tissue penetration of RF energy, combined with superior catheter stability facilitated by RMN are a possible explanation of the superior outcomes of RMN-guided ablation in general, as well as in ablation of septal and parahisian located AP’s in specific.

### 4.3 Procedure times and fluoroscopy doses

One of the major arguments against RMN in the past was the extra time needed for patient positioning and system set up, which lengthened procedure times [27]. This study observed the shortest procedure times in both RF techniques. In our opinion, RMN’s facilitated catheter navigation, combined with an experienced and dedicated RMN team, resulted in efficient RMN guided ablation procedures. Effects of radiation exposure are major for children, as for instance they are ten times more vulnerable than adults to the induction of cancer by external radiation exposure [28]. Prior studies have demonstrated lower fluoroscopy times and dosages in favor of RMN [11]. Over time, significant improvement of procedure and radiation dosages in RMN have been observed which can be attributed to the use of EAM and technological advances [27], [29]. This study observed the lowest fluoroscopy dosages in RMN and MAN AVNRT ablation, but only a tendency towards lower fluoroscopy dosages in RMN guided AVRT ablation. Further prospective research is needed to clearly define this.

### 4.4 Cost-effectiveness

Installation of the RMN technology requires an investment both in the essential equipment and specific training of the EP lab staff. In adult AVNRT ablation, Berman et al. evaluated clinical and direct cost perspectives of AVNRT ablation using either RMN, conventional manual RF and CRYO techniques [30]. RMN and conventional manual RF appeared to be equally effective and associated with lower AVNRT recurrence rates when compared to CRYO. This study observed significant disposable cost savings of conventional MAN when compared to RMN, despite similar efficacy [30]. Whether the improved outcomes of RMN in pediatrics observed by the current study, justify the additional costs of RMN over conventional manual RF techniques in AVNRT ablation remains a topic of ongoing debate.

### 4.5 Limitations

The present study's retrospective nature and the lack of blinded adjudication might have introduced bias, although this was alleviated by the use of objective measures. The large time window of inclusion might have introduced bias as techniques and approaches changed over time, however this is counterbalanced by the large numbers of inclusion of the present study, which limits its influence on the main outcomes. Principally, the large inclusion period highlights the scarcity of rhythm disorders and ablation procedures in children. In addition, we analyzed the utilization of the various ablation techniques over time and observed that all study groups included patients from the early years as well as from more recent years. However, because of technical reasons the RMN system was not operational from 2012 to 2014 in our center, which may have biased our results. Regarding AVRT, the distribution of cryoablation was skewed towards this technique being more frequently used for ablation of septal and parahisian AP’s than other locations. Nevertheless, in the subanalysis of these specific pathways, other techniques were equally frequently used to treat AP’s at these more challenging ablation sites and the primary outcomes and multivariable Cox regression analysis results were consistent with our overall findings and showed RMN to be an independent predictor of long-term freedom of recurrence.

In conclusion, in pediatric patients without structural heart disease who underwent their first ablation for AVNRT or AP-mediated tachycardia, RMN has the most favorable long-term outcomes, in addition to favorable fluoroscopy doses and procedure times.

## Declaration of Competing Interest

The authors declare that they have no known competing financial interests or personal relationships that could have appeared to influence the work reported in this paper.
