# Supplementary material for: Remote magnetic navigation shows superior long-term outcomes in pediatric atrioventricular (nodal) tachycardia ablation compared to manual radiofrequency and cryoablation
Source: Int J Cardiol Heart Vasc. 2021 Oct 1;37:100881. doi: 10.1016/j.ijcha.2021.100881 (PMC8495098; doi:10.1016/j.ijcha.2021.100881)
Supplement: Supplementary data 1 [file mmc1.docx]

**Supplemental Figure 1: Distribution of ablation techniques over time**

|  | 2008 | 2009 | 2010 | 2011 | 2012 | 2013 | 2014 | 2015 | 2016 | 2017 | 2018 | 2019 | | |
| --- | --- | --- | --- | --- | --- | --- | --- | --- | --- | --- | --- | --- | --- | --- |
| RMN | 6% | 6% | 7% | 5% | * | * | * | 4% | 23% | 15% | 22% | 12% |  |  |
| MAN | 4% | 1% | 11% | 9% | 19% | 13% | 22% | 12% | 5% | 1% | 2% | 1% |  |  |
| CRYO | 5% | 5% | 13% | 20% | 18% | 10% | 10% | 10% | 3% | 3% | 3% | 0% |  |  |

Figure 2 presents the distribution of the utilized ablation techniques over time. Percentages represent the number of procedures out of total procedures of within every given ablation technique.

* From 2012 until 2014 the RMN system was temporarily not available in our center because of technical reasons (reinstallation).
