# Supplementary material for: Remote magnetic navigation shows superior long-term outcomes in pediatric atrioventricular (nodal) tachycardia ablation compared to manual radiofrequency and cryoablation
Source: Int J Cardiol Heart Vasc. 2021 Oct 1;37:100881. doi: 10.1016/j.ijcha.2021.100881 (PMC8495098; doi:10.1016/j.ijcha.2021.100881)
Supplement: Supplementary data 2 [file mmc2.docx]

**Supplemental Figure 2: Distribution of Age and Weight**

**
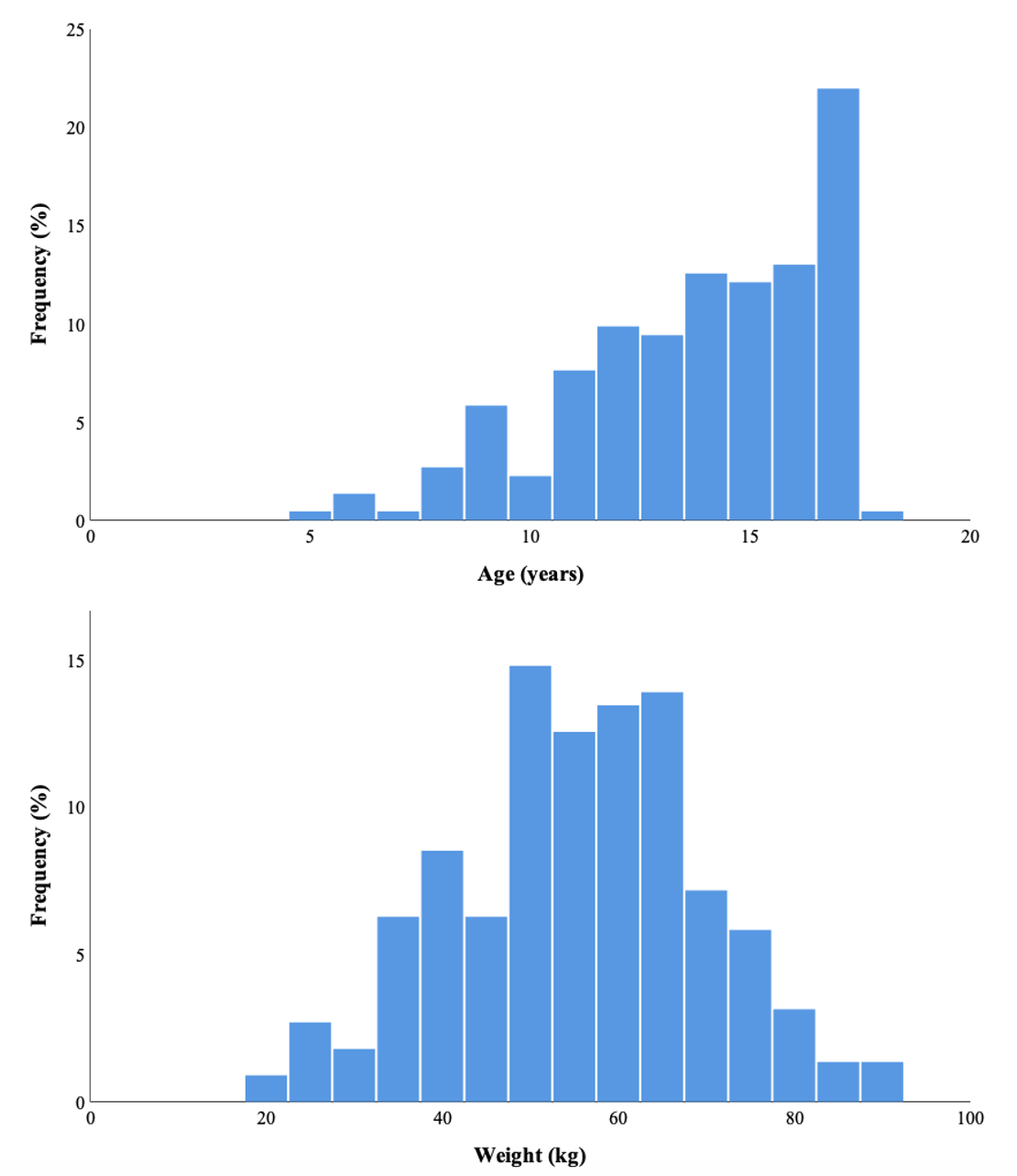
**

The distribution of Age and Weight of patients included in this study.
